# Plasma Surface Functionalization of Carbon Nanofibres with Silver, Palladium and Platinum Nanoparticles for Cost-Effective and High-Performance Supercapacitors

**DOI:** 10.3390/mi10010002

**Published:** 2018-12-21

**Authors:** Zelun Li, Shaojun Qi, Yana Liang, Zhenxue Zhang, Xiaoying Li, Hanshan Dong

**Affiliations:** School of Metallurgy and Materials, University of Birmingham, Birmingham B15 2TT, UK; zl381@cam.ac.uk (Z.L.); S.Qi@bham.ac.uk (S.Q.); YXL452@student.bham.ac.uk (Y.L.); z.zhang.1@bham.ac.uk (Z.Z.); X.li.1@bham.ac.uk (X.L.)

**Keywords:** carbon nanofibres (CNFs), active-screen plasma sputtering (ASPS) technology, supercapacitors (SCs), silver (Ag), platinum (Pt) and palladium (Pd) nanoparticles

## Abstract

Due to their relatively low cost, large surface area and good chemical and physical properties, carbon nanofibers (CNFs) are attractive for the fabrication of electrodes for supercapacitors (SCs). However, their relatively low electrical conductivity has impeded their practical application. To this end, a novel active-screen plasma activation and deposition technology has been developed to deposit silver, platinum and palladium nanoparticles on activated CNFs surfaces to increase their specific surface area and electrical conductivity, thus improving the specific capacitance. The functionalised CNFs were fully characterised using scanning electron microscope (SEM), energy dispersive X-ray analysis (EDX) and X-ray diffraction (XRD) and their electrochemical properties were evaluated using cyclic voltammetry and electrochemical impedance spectroscopy. The results showed a significant improvement in specific capacitance, as well as electrochemical impedance over the untreated CNFs. The functionalisation of CNFs via environmental-friendly active-screen plasma technology provides a promising future for cost-effective supercapacitors with high power and energy density.

## 1. Introduction

The enormous growth of electric vehicles and portable electronic devices has boosted the needs for energy storage devices simultaneously with high power and energy density. The most common energy storage devices are batteries, fuel cells and supercapacitors (SCs). The latter have attracted significant attention and research progress due to their advantageous properties over the formers. SCs exhibit greater power density satisfying the increasing demand for high power electrical appliances and fast charging, and they can withstand larger numbers of charge-discharge cycles [1,2]. However, SCs suffer from lower energy densities, high costs of raw materials and manufacturing which limit their widespread use and commercialisation [3,4,5,6,7,8,9,10,11]. One of the most crucial and greatest challenges to achieve these targets is indeed control of nanoscale materials and structures used for the SCs [12,13,14]. A large quantity of processing methods have been presented to fabricate SC electrodes based on carbon materials [15], especially carbon nanoparticles, graphene and carbon nanotubes (CNTs) [16,17]. In the past few years, two-dimensional (2D) nanomaterials including graphene, graphene-like materials, such as MXenes and transition-metal dichalcogenide (TMDs) have been explored to develop supercapacitors with enhanced electrochemical performance [18,19,20,21,22,23,24,25].

The energy storage in SCs is based on the electrostatic forces in the formation of an electrochemical double layer (EDL) between electrons and ions as well as fast redox reaction. Theoretically, the energy density of a supercapacitor is proportional to the specific capacitance and the operating cell voltage, given below [1,26,27]

(1) $$E_{s}=\frac{1}{2}C_{s}\Delta V^{2}$$

where *E_s_* is the energy density, *C_s_* is the specific capacitance, and Δ*V* is the operating cell voltage. Therefore, improved energy density can be achieved through increasing specific capacitance and extending operating cell voltage. According to Equation (2), specific capacitance of an electrode is dependent of the surface area of the electrode [1], therefore materials with a high surface area are preferable for electrode fabrication.

(2) $$C_{s}=\frac{\varepsilon A}{md}$$

where *ɛ* is the electrolyte dielectric constant, *A* is the surface area of the electrode material, *d* is the effective thickness of the EDL, and *m* is the mass of the electrode material. Also, it is crucial to retain high power density when improving energy density. Power density of supercapacitors is associated with the rate capability of electrodes which reflects how fast charge/discharge cycling can be when good capacitive behaviour is maintained. Rate capability of electrode materials is related with their electrochemical impedance, and hence low electrochemical impedance is also demanded for electrode materials [28].

Carbon materials such as carbon nanotubes (CNTs) and carbon nanofibres (CNFs) are considered as good anode materials for SCs due to their accessibility, chemical inertness in different solutions, easy processability and good temperature tolerance [29,30,31,32]. Also, many physical and chemical active methods allow for production of the material with an improved surface area and a controllable pore structure [29,32,33]. Although CNTs show unique tubular porous structures and prominent electrical properties [15], their applications are limited by the production costs [32]. Instead, inexpensive CNFs can be easily manufactured using different methods such as electrospinning [34,35,36] or vapor growth [37,38]. Also, CNFs are easy to disperse, process and functionalise achieving high conductivity and improved surface area. Therefore, CNFs have attracted great interest for making cost-effective supercapacitor electrodes. It was found that electrical conductivity of CNT films could be increased following the deposition of metallic nanoparticles such as gold, silver, platinum and palladium on CNTs by plasma sputtering [18]. Active-screen plasma sputtering technology offers a green and easy route to achieve the deposition of metallic nanoparticles [39,40,41,42], compared to electrochemical [43,44] and electroless deposition [45,46] because the former is a physical deposition method which does not involve disposal of polluting wastes or chemicals.

This work aimed at functionalising the inexpensive CNFs by coating with Ag, Pt and Pd nanoparticles via the environmentally-friendly active-screen plasma sputtering and hence improving their electrochemical properties. The results showed that the specific capacitance and electrochemical impedance of CNFs were improved significantly and good cyclability was achieved.

## 2. Materials and Methods

### 2.1. Sample Preparation

CNFs were supplied by Sigma-Aldrich Company Ltd. (Dorset, UK) and they were pyrolytically stripped. The carbon content is more than 98% with trace amounts of sulphur, calcium, silicon, nickel, chromium, sodium, magnesium and iron. Commercial CNF powders (Sigma-Aldrich) (5 g) were dispersed in isopropanol (20 mL), provided by Struers (Catcliffe, UK), to form a suspension which was then sonicated at a power of 200 W for 1 h to disperse the CNFs. The suspension was distributed into glass Petri dishes (54 mm dimeter) and left to dry overnight, allowing the CNFs attaching to the Petri dishes.

### 2.2. Active-Screen Plasma Sputtering (ASPS)

**Figure 1:**
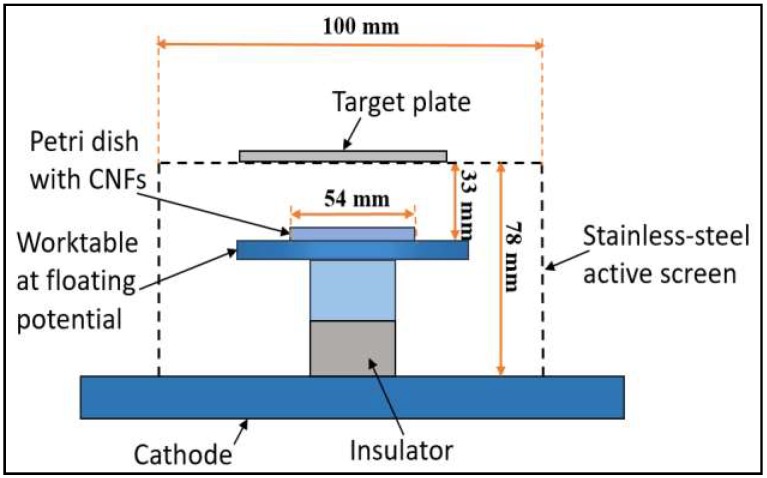
Schematic diagram of the active-screen plasma sputtering (ASPS) setup inside the plasma furnace.

The setup of active-screen plasma sputtering (ASPS) is demonstrated in Figure 1. The Petri dishes including CNFs were introduced in the vacuum chamber of a laboratory scale modified DC furnace (Klöckner Ionon GMBH, Bergisch Gladbach, Germany) with different targets of silver, palladium and platinum (specification: 80 mm × 80 mm × 0.2 mm; purity: 99.99%; Birmingham Metal Co. Ltd., Birmingham, UK), respectively. The distance between target plates and Petri dishes was 33 mm. ASPS was carried out at 320 °C at a heating rate of 500°/h in an atmosphere containing 75% hydrogen and 25% argon at a pressure of 0.75 mbar. The treatment time ranged from 0.1 to 1.0 h. The sample codes and treatment details are listed in Table 1.

### 2.3. Fabrication of Electrodes

**Figure 2:**
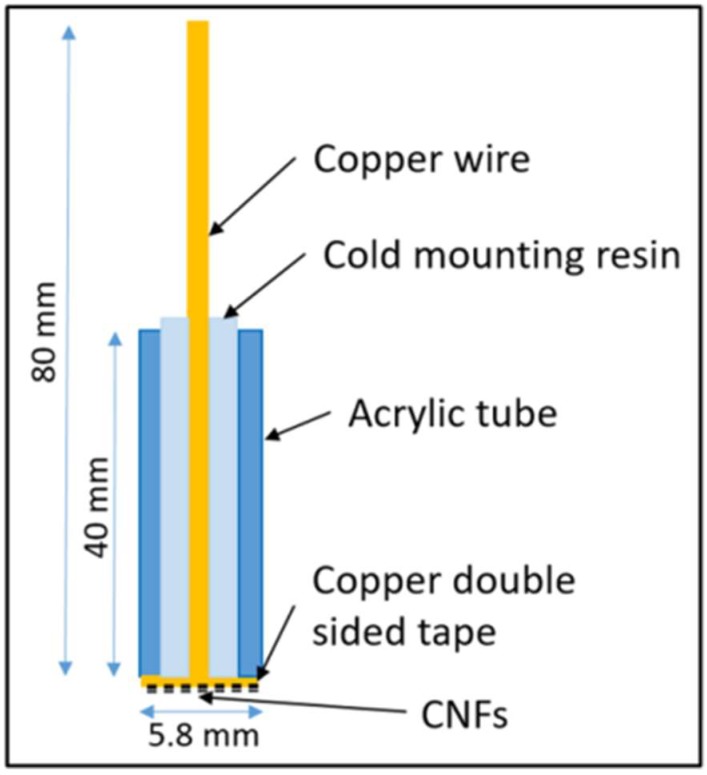
Schematic diagram of the electrodes.

The structure of the home-made working electrode is shown in Figure 2. A copper wire was cold mounted in a tube using mixture of epoxy resin and epoxy resin hardener. The resin was then ground down until the end of the copper wire was fully exposed. A disc of conductive double-sided copper tape was attached to the end. Before the electrochemical experiment, different CNFs were attached to different conductive tapes, respectively. The weight of the attached CNFs (*m*) was measured and used to calculate their specific capacitance.

### 2.4. Microstructure Characterisation

Joel 7000 scanning electron microscope (SEM) (Joel, Tokyo, Japan) equipped with an Oxford Inca energy dispersive X-ray spectroscopy (EDX) (Oxford Instruments, Abingdon, UK), was utilised to observe the morphology and collect the elemental information of deposited nanoparticles on CNFs. The phases of nanoparticles were identified using Bruker D8 ADVANCE diffractometer.

### 2.5. Electrochemical Test

Home-made three-electrode cell consisting of a working electrode (CNFs), a saturated calomel electrode (SCE) and a platinum inert counter electrode was designed for half-cell measurement. The solution was Na_2_SO_4_ (anhydrous, 99%) in de-ionized water at concentration of 1 M. The tests were carried out at room temperature. Cyclic voltammetry (CV) cycled between −0.2 and −0.7 V with respect to the SCE at different charge/discharge linear scan rates (i.e. 10, 25, 50, 100 and 200 mV/s) unfolded the charging and discharging performance of the working electrode. Cyclic voltammograms were obtained to calculate the specific capacitance of the untreated and functionalised CNFs by Equation (3) [47]. The first few cycles were neglected due to unstable initial conditions caused by electrode activation [48].

(3) $$C_{s}=\frac{Q}{2m\Delta V}=\frac{\frac{\int IdV}{v}}{2m\Delta V}=\frac{\frac{\int \frac{I}{m}dV}{v}}{2\Delta V}$$

where *C_s_* and *Q* are the specific capacitance of material and total charge in one cycle under a specific potential scan rate (*v*), respectively, *I* is the measured current through the circuit, *V* is the applied potential (vs. SCE), *∫IdV* is the integral area of a CV curve integrating the forward and backward scans in the cyclic voltammogram, Δ*V* is the operating cell voltage, *J* is the measured current density.

Electrochemical impedance spectroscopy (Nyquist diagrams) over a frequency range of 0.05 to 100,000 Hz around the open circuit potential with an alternating current (AC) perturbation of 10 mV revealed the relationship between imaginary and real impedances (*Z*_|imag|_ and *Z*_|real|_). CNFs/Pd-0.5h, CNFs/Pt-0.5h and CNFs/Ag-1.0h were then charged and discharged at a constant current density of 2, 0.8 and 0.8 A/g, respectively, for 2000 cycles to test their cyclability in terms of capacitance retention.

## 3. Results

### 3.1. Microstructure

**Figure 3:**
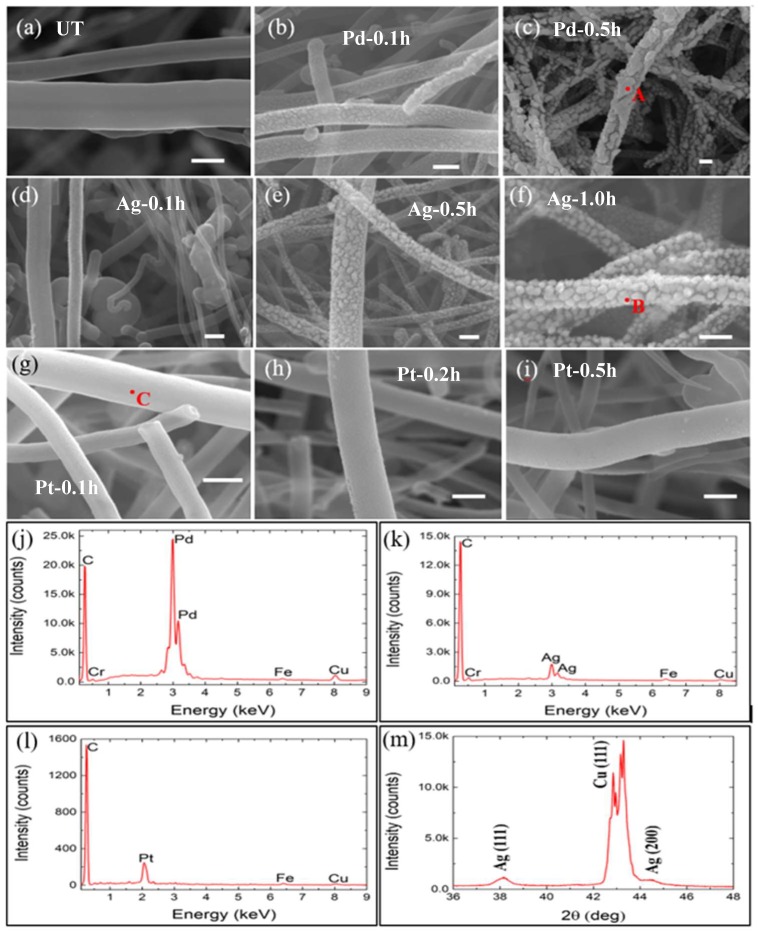
SEM images of: (**a**) untreated CNFs; (**b**) CNFs/Pd-0.1h; (**c**) CNFs/Pd-0.5h; (**d**) CNFs/Ag-0.1h; (**e**) CNFs/Ag-0.5h; (**f**) CNFs/Ag-1.0h; (**g**) CNFs/Pt-0.1h; (**h**) CNFs/Pt-0.2h; (**i**) CNFs/Pt-0.5h. All scale bars are representing 300 nm. EDX spectrum of: (**j**) CNFs/Pd-0.5h taken from spot ‘A’, denoted in (**c**); (**k**) CNFs/Ag-1.0h taken from spot ‘B’, denoted in (**f**); (l) CNFs/Pt-0.1h taken from spot ‘C’, denoted in (**g**). (**m**) XRD pattern indicating the phase of CNFs/Ag-0.5h.

Figure 3a–i show the typical morphology of the untreated CNFs and functionalised CNFs. Typical diameters of the CNFs range from 150 to 450 nm. The untreated CNFs have a smooth surface, while the functionalised CNFs have relatively rough surfaces. The EDX results display the chemical composition of the nanoparticles deposited on CNFs. The existence of silver, palladium and platinum elements was detected in functionalised CNFs, respectively. Traces of copper were observed due to the copper tape for SEM sample preparation, and traces of iron and chromium were also identified, which mainly come from the sputtering effect of the active-screen due to plasma bombardment [40,49]. Figure 3b–f show that the size of Ag and Pd nanoparticles increased with the sputtering time. The size of the Pd nanoparticles was generally greater than that of Ag nanoparticles after the same sputtering times (0.1 h and 0.5 h, respectively). The particle size of Pd reached up to 250 nm after sputtering for 0.5 h, while that of Ag was around 100 nm after sputtering for 1.0 h. The morphology of the nanoparticles appeared to change from spherical to more nodular with increasing sputtering time. The surface morphology of CNFs/Pd became inhomogeneous after sputtering for 0.5 h, whereas the distribution of the Ag nanoparticles was relatively uniform even after sputtering for 1.0 h. In comparison, the surface morphology of the CNFs/Pt was quite different (Figure 3g–i). The size of the platinum nanoparticles deposited on CNFs was very small and almost remained constant with the sputtering time. The CNFs/Pt achieved a smooth surface finish and a homogeneous as well as fine particle distribution. In addition, there were valleys between the nanoparticles, and the size of these valleys differed with nanoparticle types and sputtering time. For CNFs/Ag and CNFs/Pd, the size of valleys increased with increasing sputtering time, with the valleys becoming large as tens of nanometres after 1.0 and 0.5 h, respectively, whereas the CNFs/Pt-0.5h displayed valleys of negligible sizes and remained constant. XRD characterisation revealed equilibrium phase of the deposited element and one of the samples is shown in Figure 3m. It can be seen that the major peaks of pure silver were identified, indicating the existence of pure silver instead of silver compounds. The peaks of copper were due to the copper tape background.

### 3.2. Electrochemical Performance

**Figure 4:**
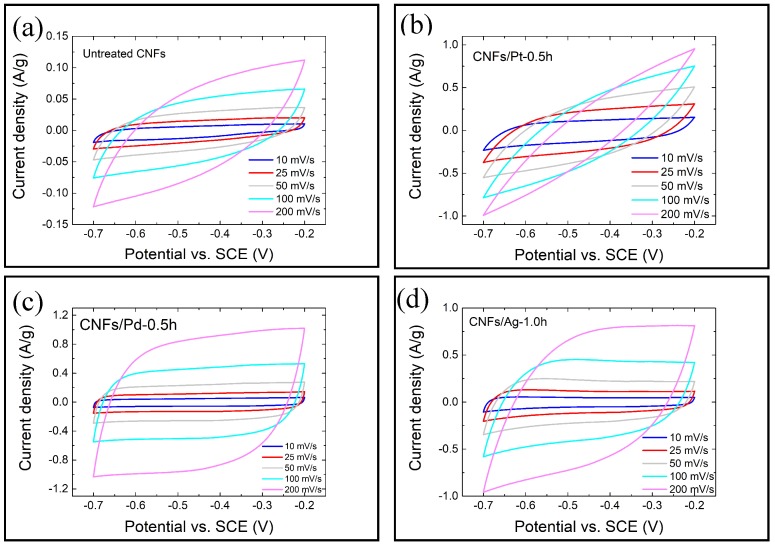
Typical cyclic voltammograms for electrodes made of: (**a**) untreated CNF; (**b**) CNFs/Pt-0.5h; (**c**) CNFs/Pd-0.5h and (**d**) CNFs/Ag-1.0h.

Figure 4a–d show typical CV curves of untreated CNFs and functionalized CNFs. The data was taken after 10 cycles. At low scan rates (10 and 25 mV/s), all CV curves exhibited quasi-rectangular shapes without obvious redox peaks, demonstrating an appropriate capacitive behavior [50]. However, the distortion from a rectangular shape became more pronounced with increasing scan rates. At high scan rates (100 and 200 mV/s), the CV curves of all samples except for CNFs/Ag-1.0h and CNFs/Pd-0.5h exhibited considerably huge distortion, indicating poor rate capability of the system [51]. This might attribute to the limited ion-penetration diffusion into the materials, where only the outer surface was in use for charge storage [52]. In comparison, CNFs/Ag-1.0h and CNFs/Pd-0.5h exhibited good rate capability [51].

**Figure 5:**
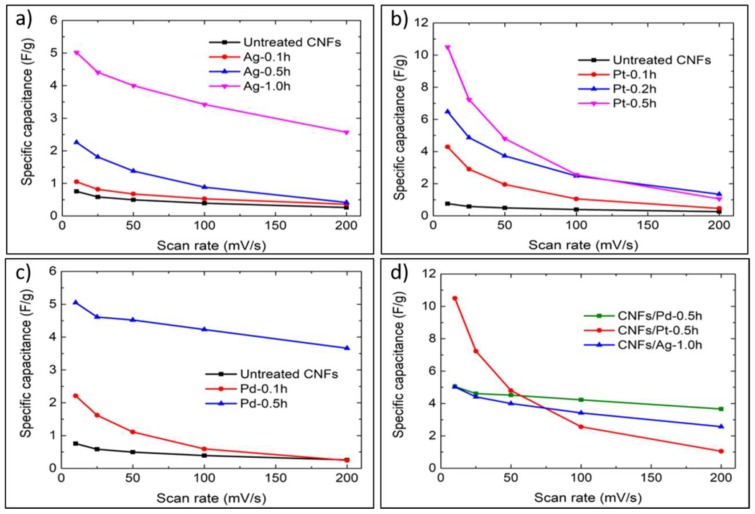
Specific capacitances for: (**a**) CNFs/Ag series; (**b**) CNFs/Pt series; (**c**) CNFs/Pd series; (**d**) CNFs/Ag-1.0h, CNFs/Pt-0.5h and CNFs/Pd-0.5h, at a series of scan rates (10 to 200 mV/s).

Figure 5a–c show that at same scan rates, the specific capacitances of functionalised CNFs were all greater than that of untreated CNFs, and they increased with the sputtering time. The specific capacitances decreased as the scan rate increased, with CNFs/Pt showing the greatest shrinkage. CNFs/Pt-0.5h, CNFs/Pd-0.5h and CNFs/Ag-1.0h exhibited the best performance within their own groups, and their values were 12.9, 5.7 and 5.6 times larger than that of untreated CNFs at 10 mV/s, respectively, and 3.0, 13.0 and 10.9 times larger at 200 mV/s. Figure 5d illustrates that the specific capacitance of CNFs/Pt-0.5h declined dramatically with scan rates, losing approximately 90% of its capacity at 200 mV/s compared with that at 10 mV/s, whilst CNFs/Ag-1.0h and CNFs/Pd-0.5h experienced less reductions in capacity as the scan rate increased. The specific capacitance for CNFs/Pd-0.5h only decreased by 27%, indicating its good rate capability [53].

**Figure 6:**
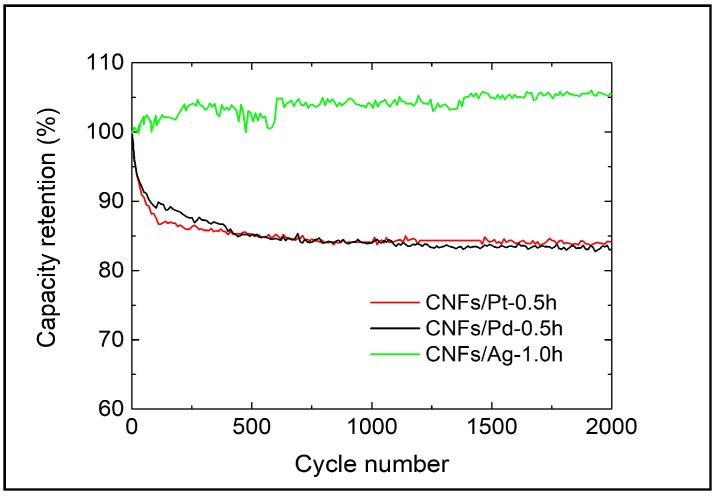
Capacitance retention of CNFs/Pd-0.5h, CNFs/Pt-0.5h and CNFs/Ag-1.0h.

In Figure 6, the capacity of CNFs/Pt-0.5h and CNFs/Pd-0.5h decreased by 15.8% and 16.9% of the initial values, respectively, over 2000 cycles. CNFs/Ag-1.0h fluctuates around the initial value. The capacity of qualified SCs commonly falls by less than 20% of the initial value within their lifetime [54]. The average charging time is: 1.30 s for Ag-1.0h at current density of 0.8 A/g, 1.40 s for Pt-0.5h at 0.8A/g, 0.77 s for Pd-0.5h at 2A/g. Therefore, the results demonstrated that CNFs/Ag-1.0h, CNFs/Pt-0.5h and CNFs/Pd-0.5h all exhibited good cycle stability for use in at least five years if they were charged and discharged once a day, and the former had the best performance.

**Figure 7:**
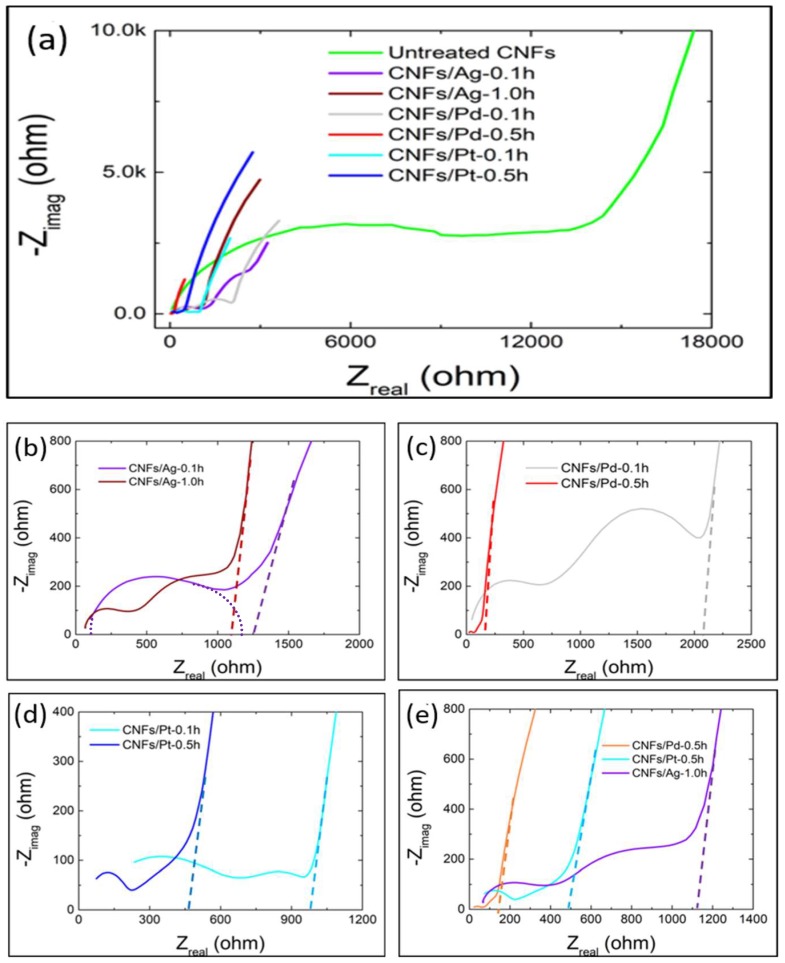
Nyquist plots of: (**a**) untreated and functionalised CNFs; (**b**) CNFs/Ag series; (**c**) CNFs/Pd series and (**d**) CNFs/Pt series and (**e**) CNFs/Ag-1.0h, CNFs/Pd-0.5h and CNFs/Pt-0.5h.

In Nyquist plots, the intersection of the projected dash lines and the *x*-axis indicates the equivalent series resistance (ESR) of the electrodes, which is associated with the electrode porous structure and determines their rate capability [5,55]. The intersections between the semicircle and the *x*-axis at lower and higher *Z*_|real|_ (e.g., in Figure 7b) represent the equivalent internal resistance (*Rs*) and charge transfer resistance (*Rct*) of the electrode material, respectively [34]. *Rs* involves the resistance of the electrode material and electrolyte, as well as the contact resistance at the interface between current collector and electrode material. *Rct* are related with the ion and electron transfer process during the formation of EDL. Figure 7a shows that all functionalised CNFs were shifted to the left and achieved smaller semicircle diameters than untreated CNFs, indicating lower charge transfer resistance of the functionalised CNFs [34]. Also, the slopes of functionalised CNFs were steeper than that of the untreated CNFs, indicating that functionalised CNFs can achieve faster EDL formation which leads to higher rate capability and hence higher power density [35,36]. Figure 7b–d show that the *Rct* and ESR for each kind of materials both decreased with increasing sputtering time, indicating low electron-and-charge transfer resistance and high rate capability, respectively [28]. Figure 7e compares the best material in each group. CNFs/Pd-0.5h exhibited the smallest *Rct* and ESR, as well as the steepest slope of the diffusion tail, indicating the fastest ion diffusion and penetration in the formation of EDL [35,36]. Therefore, it can be inferred that CNFs/Pd-0.5h had the best rate capability, which is consistent with the CV results.

## 4. Discussion

### 4.1. Effect of the Nanoparticle Deposition on the Electrochemical Performance of CNFs

The electrochemical performance for the functionalised CNFs displayed a significant improvement possibly due to the corresponding surface structures [28,55,56]. The increase of specific capacitance for functionalised CNFs may have attributed to the porous structures. In the SEM images, the valleys existing between the nanoparticles can be considered as porous structures contributing to increased surface area for ion adsorption, therefore improving the specific capacitance. Pd and Ag nanoparticles tended to agglomerate to form larger nanoparticles, whereas Pt nanoparticles piled up evenly on the previous nanoparticles. Consequently, the pore sizes of CNFs/Ag-1.0h and CNFs/Pd-0.5h were in the range of tens of nanometres while that of CNFs/Pt-0.5h was only a few nanometres. The desirable electrode materials present a hierarchical porous structure including macropores (>50 nm) for accommodating ions, mesopores (2–50 nm) for facilitating the ion transport, and nanopores (<2 nm) for boosting the charge storage [57,58]. The accessibility of ions from electrolyte into nanopores (<2 nm) depends on both the ion size and the pore dimension. It is believed that maximum EDL capacitance is yielded when the pore size is approximate to the ion size, and both larger and smaller pores cause a rapid drop in capacitance [59]. The EDL formed at CNFs is mainly due to the adsorption of hydrated Na^+^ ions with an average size of 0.358 nm [60,61]. The low specific capacitance of untreated CNFs may result from unfavourable ion adsorption due to its surface structures. In contrast, a high capacitance was obtained on CNFs/Pt-0.5h at small scan rates possibly because CNFs/Pt-0.5h achieved a large number of nanopores close to the size of Na^+^ ions. Accordingly, Na^+^ ions could access this kind of nanopore cavities and form strong interactions within the cavities, resulting in high adsorption and enhanced charge storage [62]. However, the pore size remained constant with sputtering time of up to 0.5 h, and hence, mesopores might be negligible in CNFs/Pt. The fine structure of CNFs/Pt might lead to longer paths for ion diffusion and thus higher charge transfer resistance, hindering fast formation of the EDL which in turn led to poor rate capability [36,42,43]. On the contrary, perhaps owing to the large numbers of pores equivalent to mesopores existing in their surface structures, CNFs/Pd and CNFs/Ag obtained improved rate capability with the increasing sputtering time. The relatively simple structure of mesopores could shorten the ion diffusion paths and lower the charge transfer resistance effectively, therefore promoting the ion transport and the adsorption/desorption process [58,63,64]. Their capacitances were smaller than that of CNFs/Pt-0.5h at low scan rates possibly due to the lack of nanopores [65]. The present results showed that the ASPS technology may provide a way to achieve controllable porous structures and surface properties, enabling the manipulation of electrochemical performance of SCs.

It is noted that the performance of the supercapacitors in our research was not as good as the other literature results. CNFs/Pt-0.5h showed the highest specific capacitance (~10.5 F/g) at 10 mV/s, but it was lower than the state-of-art literature results which are normally hundreds of F/g. Nonetheless, this feasibility study shows the potential of the active screen plasma treatments for the energy storage applications, and follow-on work has been planned to optimise the process and improve the performance of the final products.

### 4.2. Potential Application of the Functionalised CNFs

From the CV and EIS analysis, CNFs/Pt-0.5h exhibited the highest specific capacitance only at low scan rates, while CNFs/Pd-0.5h displayed the lowest ESR, excellent rate capability and highest specific capacitance at high scan rates. A small ESR corresponding to fast EDL formation is the key for high rate capability and high power density for SCs [1,28]. CNFs/Ag-1.0h showed lower specific capacitance and rate capability compared to CNFs/Pd-0.5h, but better cycle stability than CNFs/Pd-0.5h, which may contribute to a more consistent performance. Above all, CNFs/Pd-0.5h can be a suitable SC electrode material for short-term use, while CNFs/Ag-1.0h might be appropriate for long-term use.

## 5. Conclusions

The deposition of Ag, Pd and Pt nanoparticles on CNFs via ASPS was successful. The surface morphology of functionalised CNFs depended on target materials and sputtering time. The sizes of Ag and Pd nanoparticles both increased with sputtering time; their shapes changed from spherical to nodular, and their distribution became less uniform, whereas Pt nanoparticles remained constantly small in size. The cyclic voltammetry performance of CNFs was significantly improved after functionalisation. The specific capacitance of functionalised CNFs increased with sputtering time for each target material. CNFs/Pt-0.5h showed the highest specific capacitance (~10.5 F/g) at 10 mV/s but poor rate capability, while CNFs/Pd-0.5h and CNFs/Ag-1.0h exhibited competitive values at high scan rates. CNFs/Pd-0.5h exhibited the lowest ESR in the EIS analysis, suggesting excellent rate capability, while the excellent life-cycle was achieved on CNFs/Ag-1.0h.

## Figures and Tables

**Table 1:** Experimental conditions of active-screen plasma sputtering (ASPS).

Sample Code	Target Material	Gas	Pressure (mbar)	Temperature (°C)	Time (h)
CNFs/Ag-0.1h	Silver	25% Ar + 75% H_2_	0.75	320	0.1
CNFs/Ag-0.5h	Silver				0.5
CNFs/Ag-1.0h	Silver				1.0
CNFs/Pt-0.1h	Platinum				0.1
CNFs/Pt-0.2h	Platinum				0.2
CNFs/Pt-0.5h	Platinum				0.5
CNFs/Pd-0.1h	Palladium				0.1
CNFs/Pd-0.5h	Palladium				0.5
